# Functional neuroanatomy of spatial sound processing in Alzheimer's disease

**DOI:** 10.1016/j.neurobiolaging.2015.12.006

**Published:** 2016-03

**Authors:** Hannah L. Golden, Jennifer L. Agustus, Jennifer M. Nicholas, Jonathan M. Schott, Sebastian J. Crutch, Laura Mancini, Jason D. Warren

**Affiliations:** a Dementia Research Centre, UCL Institute of Neurology, University College London, London, UK; b Department of Medical Statistics, London School of Hygiene and Tropical Medicine, University of London, London, UK; c Neuroradiological Academic Unit, Department of Brain Repair and Rehabilitation, UCL Institute of Neurology, University, London, UK; d Lysholm Department of Neuroradiology, National Hospital for Neurology and Neurosurgery, London, UK

**Keywords:** Alzheimer's disease, Dementia, fMRI, Auditory space, Auditory scene analysis

## Abstract

Deficits of auditory scene analysis accompany Alzheimer's disease (AD). However, the functional neuroanatomy of spatial sound processing has not been defined in AD. We addressed this using a “sparse” fMRI virtual auditory spatial paradigm in 14 patients with typical AD in relation to 16 healthy age-matched individuals. Sound stimulus sequences discretely varied perceived spatial location and pitch of the sound source in a factorial design. AD was associated with loss of differentiated cortical profiles of auditory location and pitch processing at the prescribed threshold, and significant group differences were identified for processing auditory spatial variation in posterior cingulate cortex (controls > AD) and the interaction of pitch and spatial variation in posterior insula (AD > controls). These findings build on emerging evidence for altered brain mechanisms of auditory scene analysis and suggest complex dysfunction of network hubs governing the interface of internal milieu and external environment in AD. Auditory spatial processing may be a sensitive probe of this interface and contribute to characterization of brain network failure in AD and other neurodegenerative syndromes.

## 1 Introduction

“Auditory scene analysis”, the process by which we make sense of our auditory environment (Bregman, 1990), entails demanding neural computations that are performed automatically and efficiently by the normal brain. Auditory scene analysis entails the disambiguation and tracking of sound sources in space and over time, and has been shown to engage brain mechanisms in auditory association cortex in the posterior superior temporal lobe and its connections (Alain et al., 2001, Alain et al., 2008, Altmann et al., 2008, Brunetti et al., 2005, Brunetti et al., 2008, Bushara et al., 1999, Warren and Griffiths, 2003, Weeks et al., 1999, Zimmer et al., 2006). This previous evidence supports a dual organization of dorsally and ventrally directed human cortical processing streams respectively mediating sound localization and identification, and broadly analogous to the “‘what-where” dichotomy held to underpin visual object processing. The dorsal auditory stream via its inferior parietal and premotor projections is involved in preparing behavioral responses to sounds (Alain et al., 2001, Alain et al., 2008, Bushara et al., 1999, Warren et al., 2005, Weeks et al., 1999, Zimmer et al., 2006). However, auditory scene analysis is likely to involve additional cortical regions: in particular, the posterior medial cortical region (comprising posterior cingulate, precuneus and retrosplenial cortex: Leech and Sharp, 2014) has been implicated in orienting responses to auditory spatial stimuli (Bushara et al., 1999, Mayer et al., 2006, Mayer et al., 2007, Zündorf et al., 2013), whereas insula may be engaged in processing aspects of auditory motion or integrating spatial with other sound characteristics (Altmann et al., 2008, Griffiths et al., 1994, Lewis et al., 2000). Furthermore, the analysis of natural auditory scenes generally entails simultaneous processing of spatial location and identity properties of sound sources in the environment (Bregman, 1990).

Recent studies have highlighted the relationship between peripheral hearing function, cognitive performance, and regional brain atrophy (Lin et al., 2011, Lin et al., 2014). However, in addition to any peripheral hearing effect, the distributed, complex neural computations of auditory scene analysis are likely to be particularly vulnerable to the cortical pathology of Alzheimer's disease (AD). Clinical experience suggests that patients with AD often have difficulty deciphering auditory information in busy acoustic environments (Golden et al., 2015b). AD has been shown to impair various processes underpinning the analysis of auditory scenes, including segregation and binding of sound streams (Golden et al., 2015a, Goll et al., 2012), perception of sound location and motion (Golden et al., 2015b, Kurylo et al., 1993), dichotic listening and auditory attention (Gates et al., 1996, Gates et al., 2008, Gates et al., 2011, Golob et al., 2001, Golob et al., 2009, Strouse et al., 1995). Furthermore, impaired auditory scene analysis may be a harbinger of AD, manifesting presymptomatically in carriers of pathogenic mutations causing familial AD (Gates et al., 2011, Golob et al., 2009). Deficits of auditory scene analysis in AD have been correlated with alterations of gray matter structure and function in posterior lateral and medial temporo-parietal cortices that overlap the substrates of auditory spatial and pitch pattern analysis identified in the healthy brain (Brunetti et al., 2005, Golden et al., 2015b, Goll et al., 2012, Patterson et al., 2002, Warren and Griffiths, 2003, Zündorf et al., 2013). These neuroanatomical correlates include core regions of the so-called “default-mode network”: a brain network linking mesial temporal, lateral parietal, and prefrontal regions via a posterior medial cortical hub zone (Fransson and Marrelec, 2008, Raichle et al., 2001, Shulman et al., 1997) that has been identified previously as the principal target of the pathological process in AD (Buckner et al., 2005, Buckner et al., 2008, Lehmann et al., 2010, Matsuda, 2001, Minoshima et al., 1997, Scahill et al., 2002, Seeley et al., 2009, Warren et al., 2012).

In earlier work, deactivation of the default-mode network on task engagement was interpreted as evidence that this network mediates stimulus-independent thought in the resting brain (Raichle et al., 2001, Shulman et al., 1997). However, the network also participates in active processes such as imagery (Buckner and Carroll, 2007, Buckner et al., 2008, Spreng and Grady, 2010, Zvyagintsev et al., 2013) which may relate to the online representation of auditory information. The precise role of the default-mode network in these processes and more particularly the functional impact of AD on this network (and indeed, on connected brain regions beyond the putative core network) have not been defined. Previous studies using task-related fMRI in AD have focused on memory (Pihlajamäki and Sperling, 2009, Sperling et al., 2010, Sperling et al., 2003): although these studies have shown AD is associated with failure to deactivate the default-mode network normally during information encoding, it remains unclear whether this is a generic mechanism of AD-mediated dysfunction that extends to other kinds of information processing in sensory systems. Auditory scene analysis offers a clinically and anatomically relevant paradigm with which to probe AD-associated network dysfunction, whereas fMRI provides a means to assess the functional neuroanatomy of component cognitive processes and to correlate these with behavior and with structural network disintegration in AD. Previous functional neuroimaging studies assessing auditory processing in AD have been chiefly confined to the domain of memory (e.g., Dhanjal and Wise, 2014, Grossman et al., 2003a, Grossman et al., 2003b, Peters et al., 2009, Rémy et al., 2005): these studies have revealed a complex profile of AD-associated network activity shifts. In previous work, we have shown that activation of inferior parietal cortex is increased during auditory scene analysis (the “cocktail party effect”) in patients with AD relative to healthy individuals (Golden et al., 2015a). However, previous functional neuroimaging studies have not assessed the processing of sounds in space: decoding of spatial cues is fundamental to the analysis of natural auditory scenes, computationally demanding and deficient in AD (Golden et al., 2015b).

In this study, we used fMRI to assess the processing of sound sources located in space in patients with AD compared with healthy older individuals. We exploited a virtual acoustic space technique that simulates pinna filtering characteristics (Wightman and Kistler, 1989a, Wightman and Kistler, 1989b) to manipulate sound source location and pitch in a common paradigm in the scanner environment. Spatial location and pitch are both key auditory scene components, used in separating and tracking sound sources and information streams against the acoustic background (Bregman, 1990): although evidence for auditory spatial deficits in AD continues to be amassed (Golden et al., 2015b, Kurylo et al., 1993), the processing of pitch in AD may be modulated by context and in particular, whether pitch is varied within an auditory scene (Goll et al., 2011, Goll et al., 2012, Strouse et al., 1995). Although the existence of separable cortical substrates for processing pitch and spatial information has been established in the healthy brain (Warren and Griffiths, 2003), the extent of any such dichotomy in the dysfunctional cortex of AD remains unclear. Moreover, natural auditory scenes typically entail the joint processing of pitch and spatial information and these may interact (Chen et al., 2007). Accordingly, here we adopted a design in which location and pitch were varied factorially in sound sequences. In addition, we did not use an output task during scanning, as our primary interest here was to capture AD-associated alterations in obligatory, “bottom-up” brain mechanisms of spatial sound analysis, rather than task effects that might potentially be confounded by “top-down” attentional, mnestic, or effort factors; cognitive performance for processing relevant spatial sound parameters was instead assessed in post-scan behavioral testing. Based on prior cognitive and neuroanatomical evidence (Golden et al., 2015b, Goll et al., 2012), we hypothesized that AD would be associated with obligatorily altered cortical signatures of spatial sound analysis relative to healthy individuals, with loss of normal functional differentiation for the processing of pitch and spatial sound attributes. More specifically, we hypothesized a functional neuroanatomical correlate of this AD effect in posterior auditory association and temporo-parietal regions previously implicated in auditory spatial analysis and converging on the default-mode network (Golden et al., 2015b, Goll et al., 2012, Lewis et al., 2000, Warren and Griffiths, 2003, Zündorf et al., 2013).

## 2 Methods

### 2.1 Participants

Fourteen consecutive patients [6 women; mean (SD) age = 69.8 (6.3)] fulfilling criteria for typical amnestic AD (Dubois et al., 2007) and 16 healthy older individuals [9 women; mean (SD) age = 70.1 (5.0)] with no past history of neurological or psychiatric illness participated. No participant had a history of clinically significant hearing loss. At the time of participation, 12 AD patients were receiving symptomatic treatment with an acetylcholinesterase inhibitor and the remaining 2 were receiving memantine. The clinical diagnosis in the patient group was corroborated by a comprehensive neuropsychological assessment and volumetric brain MRI; no patient had radiological evidence of significant cerebrovascular damage. Demographic, clinical, and neuropsychological details for all participants are summarized in Table 1. The diagnosis of AD was further supported by cerebrospinal fluid examination (ratio total tau: beta amyloid1-42 >1 in 8 of 9 cases where cerebrospinal fluid data were available).

The study was approved by the local institutional ethics committee, and all participants gave written informed consent in accordance with the guidelines laid down in the Declaration of Helsinki.

### 2.2 Assessment of peripheral hearing

Peripheral hearing was assessed in all participants using a procedure adapted from a commercial screening audiometry software package (AUDIO-CDTM, http://www.digital-recordings.com/audiocd/audio.html). This peripheral audiometry test was administered via headphones from a notebook computer in a quiet room; participants were presented with continuous tones at 1 of 5 frequencies (500, 1000, 2000, 3000, 4000 Hz) that were initially inaudible and slowly and linearly increased in intensity. The task was to press a button as soon as the participant was sure that a tone had been detected; this response time was recorded for offline analysis. Hearing was assessed in each ear in each participant.

### 2.3 Experimental stimuli and conditions

Experimental stimuli were synthesized digitally in MATLAB 2012a (The Mathworks, Inc). A series of delay-and-add functions were applied to a Gaussian noise waveform to create iterated ripple noise (Yost, 1996); this provided a broadband carrier that allowed both manipulation of perceived sound source pitch and spatial location. Perceived pitch was generated by manipulating the latency of the delay between iterations of composite noise waveforms. Perceived spatial location was generated by convolving with generic head-related transfer functions (HRTFs) that simulate the filtering effect of the pinna and have been shown to generate a robust percept of a “virtual” sound source in external space (Wightman and Kistler, 1989a, Wightman and Kistler, 1989b). Five HRTF-specific versions of the stimulus set were created, allowing approximate matching of the corresponding generic HRTF to an individual participant's gender and height (see Table S1 in Supplementary Material). All sounds were synthesized with fixed passband 500–5000 Hz with 20 ms onset-offset ramps to eliminate click artefacts.

**Fig. 1:**
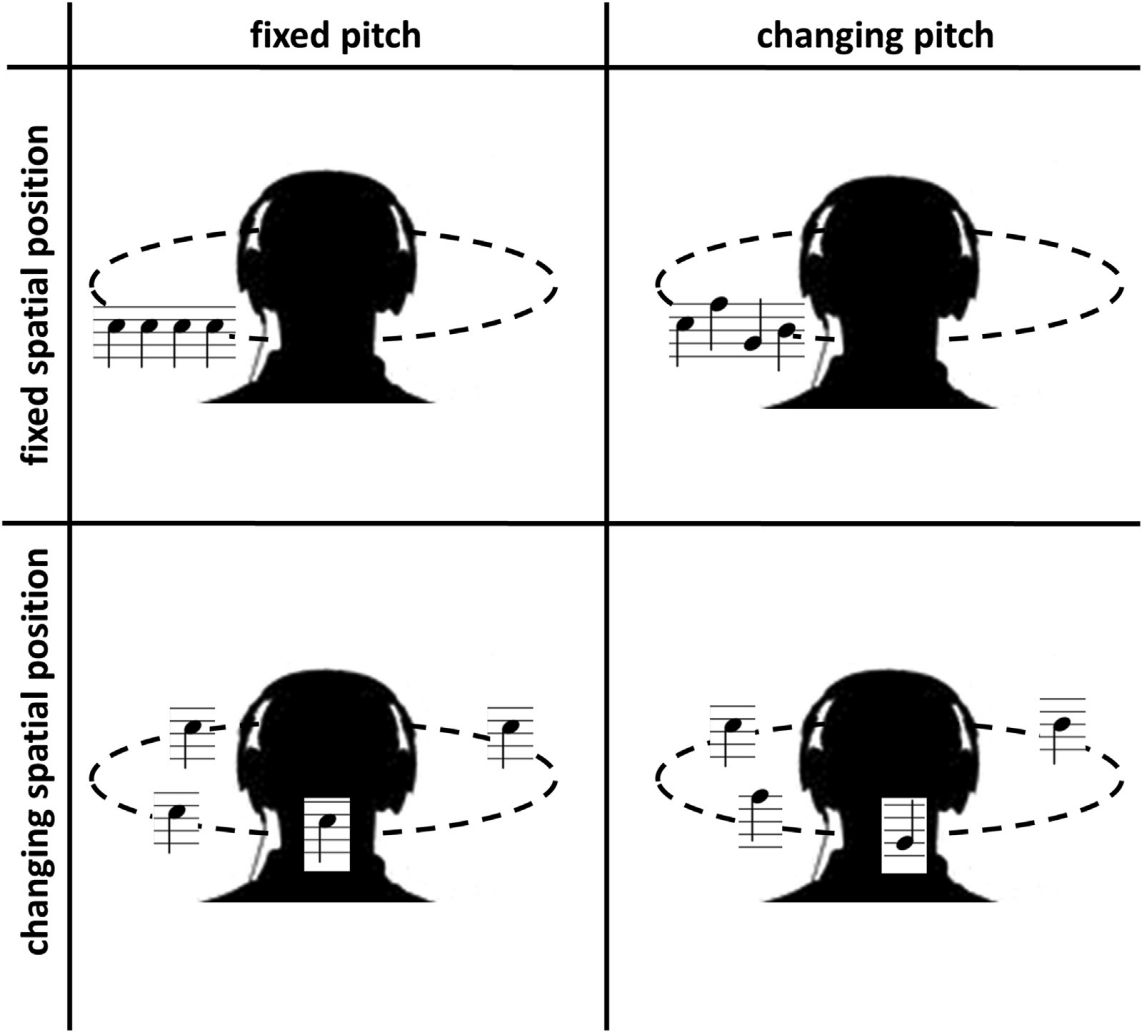
Examples of stimulus condition trials in the fMRI experiment are represented schematically (conditions were presented in randomized order during scanning). Dotted lines represent the azimuthal plane. The spatial steps and musical notation shown here are purely for illustrative purposes; stimuli were based on smaller spatial steps and pitch values that do not correspond to intervals in traditional Western music.

The experimental paradigm was adapted from previous work in the healthy young adult brain (Warren and Griffiths, 2003). Five experimental conditions were created for presentation in the scanner, as schematized in Fig. 1 (sound examples are available as Supplementary Material): (1) pitch fixed, spatial location fixed (PfSf); (2) pitch changing, spatial location fixed (PcSf); (3) pitch fixed, spatial location changing (PfSc); (4) pitch changing, spatial location changing (PcSc); and (5) silence. To create the sound conditions, individual iterated ripple noise elements of duration 300 milliseconds were concatenated with intersound pauses of duration 75 milliseconds to generate sound sequences each containing 21 elements with overall duration 7.8 seconds. For a given trial (sound sequence), pitch was either fixed or varied randomly between elements of the sequence with values 70, 85, 100, 115, 130, or 145 Hz, not corresponding to intervals in Western music; and spatial location was either fixed with starting position −90°, 0°, 90°, or 180° or randomly varied with spatial step size and direction ±30°, 40°, or 50° in azimuth, such that the initial and final elements were always identical. This generated a percept of a sound source with constant or randomly varying pitch that either repeated at the same spatial location or at varying discrete locations around the head.

#### 2.3.1 Stimulus presentation

Stimulus trials were presented from a notebook computer running the Cogent version 1.32 extension of MATLAB (http://www.vislab.ucl.ac.uk/cogent_2000.php), each triggered by the MR scanner on completion of the previous image acquisition in a “sparse” acquisition protocol. Sounds were delivered binaurally via electrodynamic headphones (http://www.mr-confon.de/) at a comfortable listening level (at least 70 dB) that was fixed for all participants; 2 identical scanning runs were administered, each comprising 16 trials for each sound condition plus 8 silence trials, yielding a total of 144 trials for the experiment. Participants were instructed to listen to the sound stimuli with their eyes open; there was no in-scanner output task or visual fixation constraint, and no behavioral responses were collected.

#### 2.3.2 Brain image acquisition

Brain images were acquired on a 3-Tesla Trio MRI scanner (Siemens, Erlangen, Germany) using a 12-channel RF receive head coil. For each of the 2 functional runs, 74 single-shot gradient-echo planar image (EPI) volumes were acquired each with 48 oblique transverse slices covering the whole brain (slice thickness 2 mm, interslice gap 1 mm and 3 mm in-plane resolution, TR/TE 70/30 ms, echo spacing 0.5 ms, matrix size 64 × 64 pixels, FoV 192 × 192 mm, phase encoding [PE] direction anterior-posterior). A slice tilt of −30° (T > C), z-shim gradient moment of +0.6 mT/m*ms and positive PE gradient polarity were used to minimize susceptibility-related loss of signal and blood-oxygen-level-dependent functional sensitivity in the temporal lobes, following optimization procedures described previously (Weiskopf et al., 2006). Sparse-sampling EPI acquisition with repetition time 11.36 s (corresponding to an interscan gap of 8 seconds) was used to reduce any interaction between scanner acoustic noise and auditory stimulus presentations. The initial 2 brain volumes in each run were performed to allow equilibrium of longitudinal T1 magnetization and discarded from further analysis. A B0 field-map was acquired (TR = 688 ms; TE1 = 4.92 ms, TE2 = 7.38 ms, 3×3×3 mm resolution, no interslice gap; matrix size = 80 × 80 pixels; FoV = 192 × 192 mm; PE direction = A-P) to allow postprocessing geometric distortion corrections of EPI data due to B0 field inhomogeneities.

A volumetric brain MR image was also obtained in each participant to allow coregistration of structural with functional neuroanatomical data. The head coil was switched to a 32-channel RF receiver head coil (Siemens, Erlangen, Germany). T1-weighted volumetric images were obtained using a sagittal 3-D magnetization prepared rapid gradient-echo sequence (TR = 2200 ms; TE = 2.9 ms; matrix size 256 × 256 pixels, voxel size of 1.1 × 1.1 × 1.1 mm).

#### 2.3.3 Post-scan behavioral testing

Following the scanning session, each participant's ability to perceive the key experimental parameters of the fMRI experiment was assessed using alternative forced choice psychoacoustic procedures that assessed pitch change detection and auditory spatial location change detection. Twenty stimuli from the scanning session were used (5 for each of the 4 sound conditions). For the spatial subtest, the task on each trial was to decide whether the sounds were fixed in position or changing between positions. For the pitch subtest, the task on each trial was to decide whether the sounds were fixed or changing in pitch. It was established that all participants understood the tasks before commencing the tests; during the tests, no feedback about performance was given, and no time limits were imposed. All responses were recorded for offline analysis.

### 2.4 Analysis of fMRI data

Brain image data were analyzed using statistical parametric mapping software (SPM8: http://www.fil.ion.ucl.ac.uk/spm). In initial image preprocessing, the EPI functional series for each participant was realigned using the first image as a reference, and images were unwarped incorporating field-map distortion information (Hutton et al., 2002). The DARTEL toolbox (Ashburner, 2007) was used to spatially normalize all individual functional images to a group mean template image in Montreal Neurological Institute (MNI) standard stereotactic space; to construct this group brain template, each individual's T1-weighted MR image was first coregistered to their EPI series and segmented using DARTEL tools (New Segment), and this segment was then used to estimate a group template that was aligned to MNI space. Functional images were smoothed using a 6-mm full-width-at-half-maximum Gaussian smoothing kernel. For the purpose of rendering statistical parametric functional maps, a study-specific mean structural brain image template was created by warping all bias-corrected native space whole-brain images to the final DARTEL template and calculating the average of the warped brain images.

Preprocessed functional images were entered into a first-level design matrix incorporating the 5 experimental conditions modeled as separate regressors convolved with the standard hemodynamic response function, and also including 6 head movement regressors generated from the realignment process. For each participant, first-level *t*-test contrast images were generated for the main effects of auditory stimulation [(PfSf + PfSc + PcSf + PcSc) − silence], changing pitch [(PcSc + PcSf) − (PfSc + PfSf)], changing spatial location [(PcSc + PfSc) − (PcSf + PfSf)] and the interaction of these effects [(PcSc − PcSf) − (PfSc − PfSf)]. Both “forward” and “reverse” contrasts were assessed in each case. Contrast images for each participant were entered into a second-level random-effects analysis in which effects within each experimental group and between the healthy control and AD groups were assessed using voxel-wise *t*-test contrasts.

Contrasts were assessed at a peak-level significance threshold *p* < 0.05 after family-wise error correction for multiple voxel-wise comparisons within neuroanatomical regions of interest in each cerebral hemisphere prespecified by our prior anatomical hypotheses. These anatomical small volumes comprised anterior superior temporal gyrus regions previously implicated in processing pitch patterns (Arnott et al., 2004, Patterson et al., 2002, Warren and Griffiths, 2003) and spatial characteristics of auditory scenes: temporoparietal junction (posterior temporal lobe and angular gyrus), posterior medial cortex (posterior cingulate, precuneus, retrosplenial cortex) and insula (Arnott et al., 2004, Brunetti et al., 2005, Brunetti et al., 2008, Griffiths et al., 1994, Lewis et al., 2000, Shomstein and Yantis, 2006, Warren and Griffiths, 2003, Zündorf et al., 2013). A region that combined anterior and posterior superior temporal gyri to encompass primary and association auditory cortex was used for the contrast assessing all sound activation. Anatomical regions were derived from Oxford-Harvard cortical (Desikan et al., 2006) and Jülich histological (Eickhoff et al., 2005) maps via FSLview (Jenkinson et al., 2012) and further edited in MRICron (http://www.mccauslandcenter.sc.edu/mricro/mricron/) to conform to the study-specific template brain image; the regions are presented in Fig. S1 in Supplementary Material.

### 2.5 Analysis of structural MRI data

Structural brain images were compared between the patient and healthy control groups in a voxel-based morphometric analysis to obtain an AD-associated regional atrophy map: normalization, segmentation, and modulation of gray and white matter images were performed using default parameter settings in SPM8, with a Gaussian smoothing kernel of 6-mm full-width-at-half-maximum. Groups were compared using voxel-wise 2-sample *t*-tests, including covariates of age, gender, and total intracranial volume. Statistical parametric maps of brain atrophy were thresholded leniently (*p* < 0.01 uncorrected for multiple voxel-wise comparisons over the whole-brain volume) to capture any significant gray matter structural changes in relation to functional activation profiles from the fMRI analysis.

### 2.6 Analysis of demographic and behavioral data

Demographic data were compared between the healthy control and AD groups using 2-sample *t*-tests (gender differences were assessed using Pearson chi-square test of distribution); neuropsychological data were compared using nonparametric Wilcoxon rank-sum tests. Tone detection thresholds on audiometry screening and performance on post-scan behavioral tasks on experimental stimuli were analyzed using linear regression models with clustered, robust standard error due to nonequal variance between groups. In the audiometry analysis, the main effect of patient group was assessed while controlling for age and frequency type, as well as assessing for any interaction between group and frequency. In the analysis of post-scan behavioral data, a robust, cluster-adjusted regression model was used to test for the main effects of disease and behavioral task on proportion of correct answers while also testing for any interaction between these 2 factors. Wald tests were used to further assess effects of interactions and specific hypotheses. Spearman correlations were performed to assess any association between peak activation for specific contrast beta weights in the fMRI analysis and d-prime scores for performance on the out-of-scanner behavioral tasks for each participant group.

## 3 Results

### 3.1 General participant characteristics

Results of the analysis of demographic and behavioral data are summarized in Table 1. The patient and healthy control groups were well matched for age (t_(28)_ = 0.13, *p* = 0.89) and gender distribution (χ^2^_(1)_ = 0.15, *p* = 0.70); however, the control group had on average significantly more years of education (t_(28)_ = 2.57, *p* = 0.02); years of education was accordingly included as a covariate of no interest in subsequent analyses of behavioral data. As anticipated, the AD group performed significantly worse than the healthy control group on a range of neuropsychological measures; referenced to normative data for this age group, AD patients showed particularly severe deficits of episodic memory, executive function, naming, and visuospatial working memory. Tone detection thresholds on audiometry did not differ between the patient and healthy control groups (β = 170, *p* = 0.94, CI −4198 to 4540), nor was there any significant interaction between group and sound frequency (F_(4,29)_ = 1.11, *p* = 0.37); accordingly, peripheral hearing function was not considered further as a factor in analyses.

### 3.2 Post-scan behavioral data

Group performance data for the post-scan behavioral tests are presented in Table 1. The AD group performed significantly worse than the healthy control group on both the pitch and spatial tasks (beta = −3.32, *p* = 0.006, CI −5.60 to −1.03); scores did not differ significantly between task type (beta = −0.75, *p* = 0.193, CI −1.90 to 0.40), and there was no significant interaction between group and test type (F_(1,29)_ = 0.90, *p* = 0.35). Eight individuals with AD on the spatial task and 3 on the pitch task performed below the range of the healthy control group.

### 3.3 Structural neuroanatomical data

**Fig. 2:**
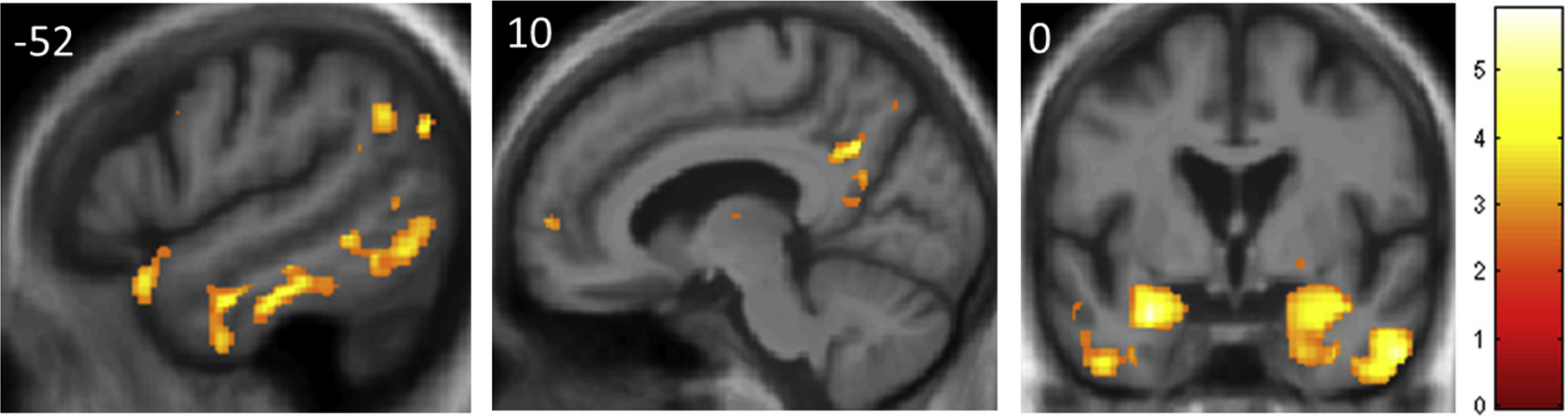
Distribution of gray matter atrophy in the Alzheimer's disease group. Statistical parametric maps of regional gray matter atrophy in the Alzheimer's disease group compared to the healthy control group from the voxel-based morphometry analysis are shown. Maps are presented on a group mean T1-weighted MR image in MNI space, thresholded leniently for display purposes at *p* < 0.01 uncorrected for multiple voxel-wise comparisons over the whole brain. The color side bar codes voxel-wise t-values of gray matter change. Planes of representative sections are indicated using the corresponding MNI coordinates (mm); the right hemisphere is shown on the right in the coronal section. (For interpretation of the references to color in this figure legend, the reader is referred to the Web version of this article.)

Comparison of the AD and healthy control groups in the voxel-based morphometric analysis revealed the anticipated profile of AD-associated regional gray matter atrophy involving hippocampi, temporal, temporoparietal, and posterior medial cortices. Statistical parametric maps are presented in Fig. 2, and further details about regional atrophy profiles with local maxima of gray matter loss are presented in Supplementary Table S2 in Supplementary Material.

### 3.4 Functional neuroanatomical data

**Fig. 3:**
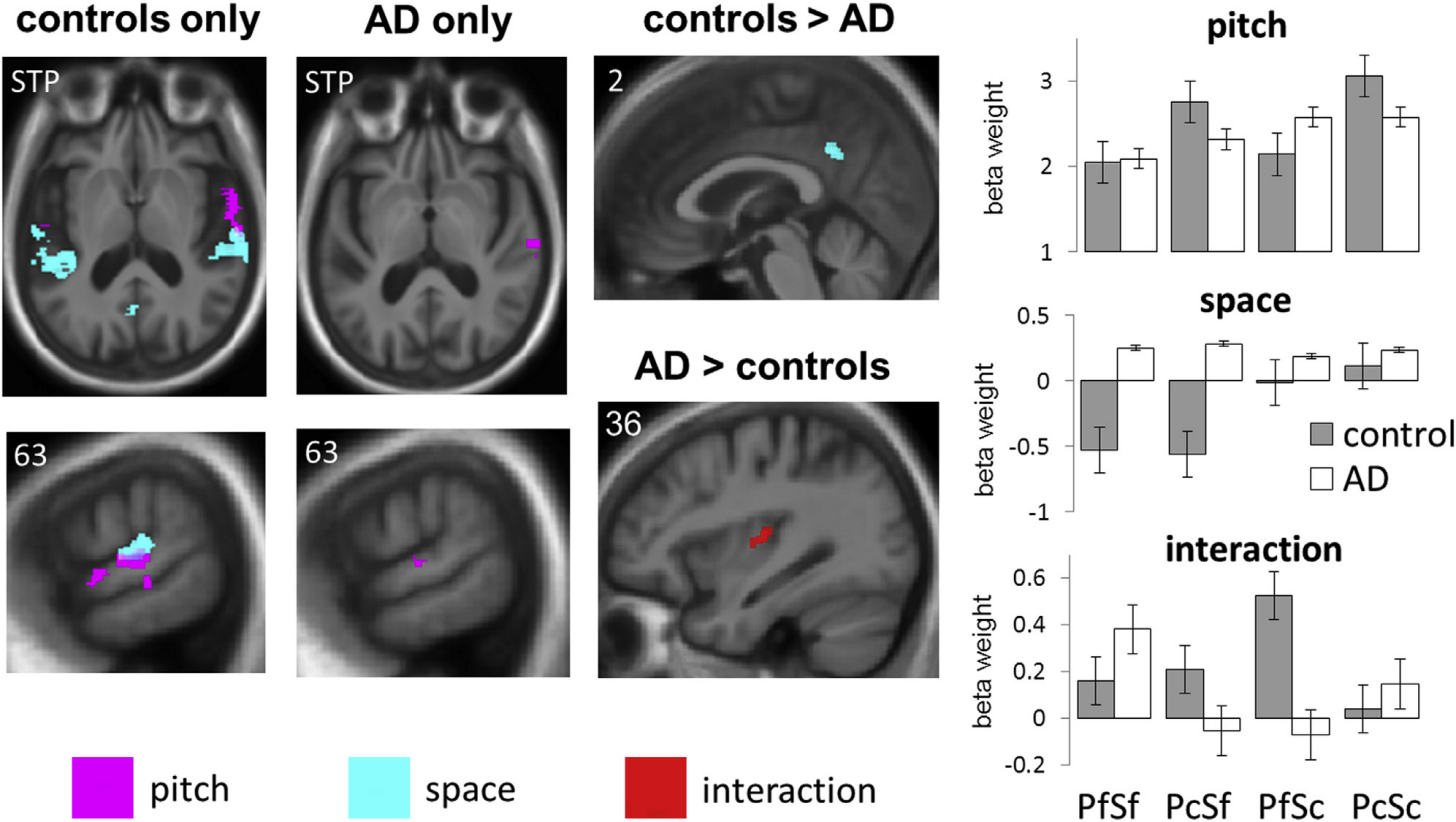
Functional neuroanatomical substrates for the analysis of spatial sounds and the effect of Alzheimer's disease (AD). Statistical parametric maps show all significant regional brain activations identified within the healthy control group (far left panels), the AD group (middle left panels), and in group comparisons (middle right panels); maps have been rendered on representative axial (top left) and sagittal sections of the study-specific group mean T1-weighted structural MR image. The MNI coordinate of each section plane is indicated (the axial section is tilted to display auditory cortical areas in the superior temporal plane (STP); the right hemisphere is shown on the right). Maps have been thresholded at *p* < 0.001 uncorrected for multiple comparisons over the whole brain, showing clusters >50 voxels, for display purposes; for healthy controls and group comparisons, clusters shown were also significant at threshold *p* < 0.05 after correction for multiple comparisons within prespecified anatomical regions of interest (see also Table 2 and Table S3). Contrasts were composed as follows: pitch variation (pitch − magenta), [(PcSc + PcSf) − (PfSc + PfSf)]; spatial variation (space − cyan), [(PcSc + PfSc) − (PcSf + PfSf)]; spatial-pitch interaction (interaction, red), [(PcSc–PcSf) − (PfSc − PfSf)]. Also shown (far right panels) are plots of beta weights (group mean ±1 standard error beta parameter estimates) at the peak voxel for the pitch variation contrast in the healthy control group (in anterior superior temporal cortex, top; not significant at the prescribed corrected threshold in the AD group), and for significant group comparisons in the spatial variation contrast (healthy control group greater than AD group in posterior cingulate cortex, middle) and the spatial-pitch interaction contrast (AD group greater than control group in posterior insula, below). Abbreviations: PcSc, pitch changing, spatial location changing; PcSf, pitch changing, spatial location fixed; PfSc, pitch fixed, spatial location changing; PfSf, pitch fixed, spatial location fixed. (For interpretation of the references to color in this figure legend, the reader is referred to the Web version of this article.)

Statistical parametric maps of significant activation for contrasts of interest are presented in Fig. 3 and in Fig. S2 in Supplementary Material; significant local maxima are summarized in Table 2 (additional activations observed at a more lenient significance threshold *p* < 0.001 uncorrected for multiple comparisons over the whole brain are presented in Table S3 in Supplementary Material). Auditory stimulation (the contrast of all sound conditions over silence) produced as anticipated extensive bilateral activation of Heschl's gyrus and superior temporal gyrus, in both the healthy control and AD groups (see Figure S2). Pitch variation (changing over fixed pitch) produced activation of right anterior superior temporal gyrus and sulcus in the healthy control group but no activation in the AD group at the prescribed threshold (activation was observed for the AD group in posterior superior temporal cortex at a relaxed uncorrected threshold; Fig. 3 and Table S3). Auditory spatial variation (changing over fixed sound location) produced bilateral activation of posterior superior temporal gyrus, planum temporale, and posterior cingulate cortex in the healthy control group but no activation in the AD group at the prescribed threshold. No significant activations were identified for the “reverse” contrasts of fixed over changing pitch or fixed over changing spatial location. The interaction of spatial and pitch variation elicited significant activation in left anterior superior temporal cortex in the healthy control group and significant activation in right posterior insula in the AD group.

When the AD and healthy control groups were compared directly, the effect of auditory spatial variation was significantly greater in the healthy control group than the AD group in posterior cingulate cortex (Fig. 3). Post hoc analysis of condition beta weights revealed that this group-wise interaction was driven by significantly higher beta values for conditions with changing versus fixed auditory spatial location (greater deactivation in conditions with fixed auditory spatial location) in posterior cingulate in the healthy control group. The interaction of auditory spatial and pitch variation produced significantly greater activation of right posterior insula in the AD group versus the healthy control group; post hoc analysis of condition beta weights for this interaction revealed no significant pairwise group or condition differences but rather, mirror beta profiles in the 2 groups (the AD group showed less activation in conditions where pitch or auditory spatial change occurred in isolation than in conditions where pitch and auditory spatial location were both fixed or changing simultaneously, whereas the healthy control group showed the reverse pattern).

The healthy control group showed a significant inverse correlation between peak activation in posterior cingulate cortex and d-prime for the auditory spatial task (r_(s)_ = −0.55, *p* = 0.03), but no significant correlations between peak activation in insula and d-prime for either task. The AD group showed no significant correlations between peak regional activations and d-prime for either task (spatial task in posterior cingulate, r_(s)_ = 0.34, *p* = 0.23; spatial task in right posterior insula r_(s)_ = 0.03, *p* = 0.93; pitch task in posterior insula, r_(s)_ = −0.38, *p* = 0.18).

## 4 Discussion

Here we have shown that functional neuroanatomical mechanisms for processing spatial sounds are altered in AD compared to the healthy older brain. Elementary sound encoding (the effect of any auditory stimulation compared with silence) produced similar activation in patients with AD and in healthy older individuals, indicating that AD targets higher order processing of sound attributes. In the older control group, the processing of sequential pitch variation activated anterior superior temporal cortex, consistent with previous evidence for pitch pattern analysis in the healthy brain (Patterson et al., 2002, Warren and Griffiths, 2003). Although this activation profile was not observed at the prescribed threshold in the patients with AD, the experimental groups did not differ significantly in the processing of pitch variation per se. In contrast, the groups did show significantly different activation profiles in response to changing sound location in posterior cingulate cortex. This was driven chiefly by failure of the normal deactivation of posterior cingulate cortex in the fixed auditory spatial location conditions in AD group (Fig. 3); AD was associated with loss of functional differentiation of posterior cingulate responses that was evident in healthy older individuals. Unlike the healthy control group, the AD group showed an interaction between pitch and spatial sequence processing in posterior insula, and this group difference was also significant. The form of this interaction was complex and driven by mirror profiles of activation in the AD and healthy control groups (Fig. 3): the normal profile of enhanced activation shown by controls in conditions with congruent compared with incongruent pitch and spatial variation was reversed in the AD group. Furthermore, functional neuroanatomical differences between the AD and healthy older control groups extended (particularly in the case of the insular interaction effect) beyond the zone of disease-associated gray matter atrophy as characterized in a parallel structural neuroanatomical comparison between the groups (Fig. 2).

Taken together, these findings suggest that AD is associated with specific functional alterations in a brain network engaged in processing spatial sounds. This study builds on previous evidence in the healthy brain demonstrating that posterior medial cortex is engaged during analysis of both spatial and nonspatial information in auditory scenes (Bushara et al., 1999, Mayer et al., 2006, Mayer et al., 2007, Wong et al., 2009, Wong et al., 2008, Zündorf et al., 2013). More particularly, the present work corroborates other evidence for dysfunction of brain mechanisms that mediate aspects of auditory scene analysis including auditory source localization in AD (Gates et al., 2008, Gates et al., 2011, Gates et al., 1996, Golden et al., 2015a, Golden et al., 2015b, Goll et al., 2012, Golob et al., 2001, Golob et al., 2009, Kurylo et al., 1993, Strouse et al., 1995). Previous studies of patients with AD have demonstrated structural gray matter correlates of sound stream disambiguation in posterior cingulate cortex and auditory spatial discrimination in precuneus (Golden et al., 2015b, Goll et al., 2012). Posterior medial cortical regions generally work in concert to mediate various aspects of self-awareness and directed attention (Leech and Sharp, 2014, Vogt and Laureys, 2005). Although the precise roles of these cortices in auditory scene analysis have not been defined, posterior medial cortex may be preferentially involved in reorienting of attention between locations in egocentric space (Mayer et al., 2006, Mayer et al., 2007, Shomstein and Yantis, 2006) or (during passive spatial listening, as here) implicit tracking of a sound source over a series of location shifts around the head. This may reflect a broader role of this cortical region in the cognition of spatial navigation (Miller et al., 2014). Moreover, posterior cingulate cortex is a hub zone of the putative default-mode network implicated as core to the pathogenesis of AD (Buckner et al., 2005, Fransson and Marrelec, 2008, Lehmann et al., 2010, Minoshima et al., 1997, Warren et al., 2012).

The role of insula in processing auditory information continues to be defined. Unlike posterior cingulate cortex, insula is not a core default-mode network (DMN) component, but it is likely to act as a multimodal hub that integrates body state information with incoming sensory traffic from the external environment: as such, this region is well placed to link DMN with brain networks that evaluate sensory stimuli and program behavioral responses, in particular the anterior fronto-insular “salience network” (Seeley et al., 2009, Zhou and Seeley, 2014). Previous work has implicated insula cortex in the analysis of sound movement particularly motion relative to self (Griffiths et al., 1997, Griffiths et al., 1994, Lewis et al., 2000); however, this multimodal region has functional subdivisions and a range of potentially relevant functions that have yet to be fully defined (Bamiou et al., 2003): these include fine-grained analysis of auditory timing cues (Bamiou et al., 2006) and the modulation of spatial-by-nonspatial auditory object features (Altmann et al., 2008). Insular activity is sensitive to cognitive load in the processing of musical and other sound patterns (Altmann et al., 2008, Nan et al., 2008) and to the detection of changes across sensory modalities (Downar et al., 2000): considered together with evidence that insula and its connections to DMN are affected relatively early in the course of AD (Xie et al., 2012), it is therefore plausible that the interaction of spatial and pitch pattern processing here should engage insular cortex in AD but not in the healthy older brain. Activity in this region was not correlated with performance on post-scan spatial or pitch discrimination tasks in the present patient cohort, suggesting that this engagement of insula in AD did not fulfill any compensatory role during auditory scene analysis. Insular involvement here might in principle reflect differential engagement of multimodal regions during processing of computationally demanding sensory traffic (e.g., calibration of a stable pitch or spatial template while the other parameter is changing); alternatively, it might represent an entirely aberrant activation profile produced by AD. One potentially unifying interpretation of the present findings might invoke dysfunctional coupling between posterior cingulate and insular cortex in AD, leading to impaired ability to update mental representations of a sound source with shifting spatial or pitch trajectories: this would be consistent with a role for posterior cingulate cortex in tuning brain network activity between internally and externally directed cognitive operations (Leech and Sharp, 2014).

This profile of cortical dysfunction in AD is unlikely to reflect simply attenuation of activity due to pathological gray matter loss. Inspection of condition effect sizes in the present healthy control and AD groups (Fig. 3) reveals complex profiles of bidirectional activity shifts in AD patients relative to healthy older individuals. In particular, posterior cingulate cortex in AD patients did not show the normal pattern of reduced activation in response to sounds with fixed versus changing spatial location. Although activity shifts are more difficult to interpret in the absence of an output task, this pattern in AD is consistent with failure to deactivate posterior cingulate cortex normally. In the healthy brain, deactivation of posterior cingulate might play a crucial modulatory or permissive role in bringing other brain areas on line during analysis of spatial sounds: this interpretation is in line with the present data (Fig. 3) which further suggest that AD leads to a loss of the normal inverse correlation between posterior cingulate activity and auditory spatial perceptual performance (as indexed here by the out-of-scanner behavioral task). An analogous failure to modulate activity in posterior medial cortex has been linked previously to impaired memory performance in AD (Celone et al., 2006, Pihlajamäki and DePeau, 2008, Pihlajamäki and Sperling, 2009, Sperling et al., 2010, Sperling et al., 2003) and may constitute a generic mechanism of AD-associated default-mode dysfunction. It is likely that dysfunction of this key hub region is modulated by connectivity with other brain regions and by tasks engaging auditory attention (Kamourieh et al., 2015). More fundamentally, it remains unclear to what extent altered BOLD signal responses may reflect the effects of AD or acetylcholinesterase inhibitor treatment on regional cerebral hemodynamic responses (Bentley et al., 2008, Rombouts et al., 2005, Thiyagesh et al., 2010).

Although this study was not primarily designed to elucidate brain mechanisms of auditory “what” and “where” processing, our findings support a functional neuroanatomical dichotomy for processing spatial and nonspatial auditory information in healthy older controls, albeit with some potential for interaction between these dimensions. Relative to healthy controls, the AD group showed comparable behavioral deficits in processing both pitch changes and location changes. Whereas pitch processing has been found to be relatively preserved relative to spatial processing in previous neuropsychological studies of AD (Golden et al., 2015b, Goll et al., 2012, Kurylo et al., 1993, Strouse et al., 1995), the stimuli used here depart from previous work in requiring conjoint processing of pitch changes in the presence of simultaneous spatial cues and over extended sound sequences. It might therefore be argued that the present stimuli more closely reflect the increased task demands imposed by natural auditory scenes, in which pitch information must be extracted (as here) from sounds in space. The lack of functional neuroanatomical differentiation between groups for the pitch processing contrast here therefore appears somewhat paradoxical but might be attributable to several factors. Care is needed, firstly, in interpreting null effects in fMRI analyses because these at least in part reflect statistical thresholding (using a more relaxed whole-brain threshold, activation was evident in the AD group for the pitch contrast though not the spatial contrast: see Fig. 3 and Table S3). In addition, the out-of-scanner behavioral tasks here were not intended to provide a detailed stratification of pitch and spatial processing impairments, which are more fully delineated using customized, graded difficulty stimuli (Golden et al., 2015b). Moreover, behavioral deficits need not have a discrete regional neuroanatomical mapping: the pitch deficit in the AD group relative to healthy controls might, for example, arise from altered network connectivity, which was not captured here. The lack of significant within-group fMRI signatures of pitch and spatial change processing in the present AD group at the prescribed threshold occurred despite a normal response to auditory stimulation per se: rather than some generalized failure of auditory cortical processing, AD may predominantly affect the processing of higher order sound attributes. The lack of significantly differentiable cortical signatures of pitch and spatial processing and their abnormal interaction in posterior insula together argue for loss of selectivity of auditory cortical mechanisms in AD. The present data extend earlier work showing that the pathological process in AD targets cortical mechanisms of auditory scene analysis (Golden et al., 2015a, Golden et al., 2015b, Goll et al., 2012), and more broadly, align with other evidence that the functional integrity of the default-mode network and interacting cortical networks is disrupted in AD (Dennis and Thompson, 2014).

This study has certain limitations that suggest directions for future work. Case numbers here were relatively small; the findings should be substantiated in larger cohorts representing AD phenotypic variants, which may have distinct auditory spatial signatures (Golden et al., 2015b) as well as non-AD dementias. Deficits of auditory scene analysis may be early markers of AD (Gates et al., 2011, Gates et al., 2002, Golob et al., 2009): this should be further assessed in longitudinal studies with fMRI correlation, ideally including presymptomatic individuals with genetic AD. The present passive listening paradigm was designed to address mechanisms of obligatory perceptual analysis. These mechanisms are likely to be modulated by output task, memory, and attentional demands (Kamourieh et al., 2015, Warren et al., 2005) and by mechanisms for coding behavioral stimulus salience that may also be altered in AD (Fletcher et al., 2015): such factors should be investigated explicitly. Related to this, the processing of spatial sounds should be assessed under more ecological conditions requiring integration of multimodal cues. Besides anatomical mapping, functional network connectivity alterations may capture additional disease effects and should be investigated directly (e.g., using graph theoretical techniques). From a more basic physiological perspective, interpretation of fMRI studies in AD will require elucidation of the impact of disease and drugs modulating cholinergic function on cerebral hemodynamic responses, defined using continuous sampling of the BOLD signal rather than the sparse “snapshots” captured in the present acquisition protocol. Taking these limitations into account, this study consolidates a growing body of work suggesting that auditory scene analysis may be a sensitive probe of brain network disintegration in AD (Golden et al., 2015a, Golden et al., 2015b, Goll et al., 2012). Tracking sound sources in space requires updating of an internal sensory image by incoming sensory information and precise dynamic coding of sensory signals: neural operations that are likely to be peculiarly vulnerable to the anatomical topography of AD (Leech and Sharp, 2014, Vogt and Laureys, 2005) and to the effects of neurodegenerative pathology on essential electrophysiological properties of cortical neurons (Ahveninen et al., 2014). Future work could test these ideas directly by comparing large-scale brain network interactions in AD and diseases (such as the frontotemporal lobar degenerations) with distinct network signatures (Zhou and Seeley, 2014); and by manipulating spatial and nonspatial attributes of more complex, naturalistic auditory “scenes,” such as music.

## Disclosure statement

The authors have no conflicts of interest to disclose.

## Figures and Tables

**Table 1:** Demographic and neuropsychological characteristics of participant groups

Characteristic	Healthy controls	AD
General demographic and clinical		
No. (m:f)	8:8	8:6
Age (yrs)	70.1 (5.0)	69.8 (6.3)
Handedness (R:L)	15:1	13:1
Education (yrs)	16.0 (2.3)	13.3 (3.4)^a^
MMSE (/30)	29 (1.1)	20 (5.1)
Symptom duration (yrs)	—	5.8 (2.0)
General neuropsychological assessment		
General intellect: IQ		
WASI verbal IQ	120 (8.9)	94 (17.2)^a^
WASI performance IQ	121 (15.7)	93 (22.2)^a^
NART estimated premorbid IQ	122 (5.5)	108 (15.6)^a^
Episodic memory		
RMT words (/50)	47 (2.2)	**31 (7.4)**^a^
RMT faces (/50)	43 (4.2)	**34 (6.9)**^a^
Camden PAL (/24)	21 (2.5)	**3.4 (3.9)**^a^
Executive skills		
WASI Block Design (/71)	43 (16.0)	19 (14.0)^a^
WASI Matrices (/32)	28 (12.5)	13 (8.4)^a^
WMS-R digit span forward (/12)	8.6 (1.9)	6.6 (1.7)^a^
WMS-R digit span reverse (/12)	7.2 (2.2)	4.7 (1.8)^a^
WMS-III spatial span forward (/16)	6.8 (1.7)	5.1 (2.2)
WMS-III spatial span reverse (/16)	6.9 (1.2)	**3.4 (2.2)**^a^
D-KEFS Stroop color (s)^b^	31 (7.3)	**53 (21.0)**^a^
D-KEFS Stroop word (s)^b^	21 (4.2)	**35 (18.1)**^a^
D-KEFS Stroop interference (s)^b^	65 (18.1)	**103 (47.9)**^a^
Letter fluency (F: total)	17 (6.0)	9 (4.9)^a^
Category fluency (animals: total)	21 (5.1)	**11 (5.0)**^a^
Trails A (s)^c^	34 (10.7)	**70 (50.3)**^a^
Trails B (s)^d^	78 (20.1)	196 (73.7)^a^
WAIS-R Digit Symbol (total)^e^	52 (10.5)	**26 (15.4)**^a^
Language skills		
WASI Vocabulary (/80)	70 (4.6)	52 (13.7)^a^
WASI Similarities (/48)	40 (6.9)	24 (12.4)^a^
GNT (/30)	26 (2.0)	**14 (7.8)**^a^
BPVS (/150)	147 (1.9)	135 (21.4)^a^
NART (/50)	43 (4.5)	34 (10.7)^a^
Posterior cortical skills		
GDA (/24)	16 (4.2)	6 (6.2)^a^
VOSP Object Decision (/20)	18 (2.2)	15 (3.7)^a^
VOSP Dot Counting (/10)	9.9 (0.3)	8.6 (1.9)^a^
Post-scan behavioral tasks		
Auditory spatial change detection (/20)	18.6 (1.3)	14.9 (3.6)^a^
Pitch change detection (/20)	17.9 (2.4)	15.6 (3.6)^a^

Mean (standard deviation in parentheses) performance scores are shown unless otherwise indicated. Maximum scores on neuropsychological tests are shown in parentheses. Results in bold indicate mean score <5th percentile for age norms (not available for BPVS and letter fluency).

Key: AD, Alzheimer's disease; BPVS, British Picture Vocabulary Scale (Dunn et al., 1982); D-KEFS, Delis Kaplan Executive System (Delis et al., 2001); GDA, Graded Difficulty Arithmetic (Jackson and Warrington, 1986); GNT, Graded Naming Test (McKenna and Warrington, 1983); NART, National Adult Reading Test (Nelson, 1982); PAL, Paired Associates Learning (Warrington, 1996); RMT, Recognition Memory Test (Warrington, 1984); VOSP, Visual Object and Spatial Perception Battery (Warrington and James, 1991); WASI, Wechsler Abbreviated Scale of Intelligence (Wechsler, 1999); WMS-R, Wechsler Memory Scale–Revised (Wechsler, 1987); WMS-III, Wechsler Memory Scale 3rd edition (Wechsler, 1997).

a Significantly different from control group (*p* < 0.05).

b 13 patients completed this task.

c 12 patients completed this subtest.

d 9 patients completed this subtest.

e 11 patients completed this task.

**Table 2:** Summary of fMRI data for auditory contrasts of interest in participant groups

Group	Contrast	Region	Side	Cluster (Voxels)	Peak (mm)			t-value	*p*-value
x	y	z
Healthy controls	Sound > silence^a^	HG/STG	L	4236	−51	−15	1	21.51	<0.001
HG/STG	R	2704	58	−27	12	9.96	<0.001
Changing > fixed pitch^b^	Anterior STG/STS	R	477	59	2	−3	7.14	0.003
Changing > fixed location^c^	PT/posterior STG	L	933	−39	−37	15	8.69	0.001
PT/posterior STG	R	584	66	−24	6	7.64	0.002
Posterior cingulate cortex	L	318	0	−48	34	6.29	0.016
Posterior cingulate cortex	R	109	2	−46	36	5.96	0.025
Changing pitch versus changing location^d^	Anterior STG/STS	L	53	−63	−12	4	6.34	0.008
AD patients	Sound > silence^a^	HG/STG	L	3301	−56	−10	−2	14.72	<0.001
HG/STG	R	2007	48	−16	4	10.18	<0.001
Changing pitch versus changing location^d^	Posterior insula	R	51	36	−16	7	7.52	0.005
Controls > AD	Changing > fixed location^c^	Posterior cingulate cortex	L	95	0	−48	34	4.51	0.049
Posterior cingulate cortex	R	56	2	−48	34	4.51	0.049
AD > controls	Changing pitch versus changing location^d^	Posterior insula	R	66	36	−16	9	4.77	0.016

Regional brain activations for contrasts between auditory conditions of interest within each participant group and between groups are shown; all associations significant at peak-level threshold *p* < 0.05_FWE_ corrected for multiple voxel-wise comparisons within prespecified anatomical regions in clusters >50 voxels in size are presented. Contrasts were composed as coded by superscripts.

Key: PfSf, fixed pitch, fixed auditory spatial location; PcSf, changing pitch, fixed spatial location; PfSc, fixed pitch, changing spatial location; PcSc, changing pitch, changing spatial location; AD, Alzheimer's disease; HG, Heschl's gyrus; PT, planum temporale; STG/S, superior temporal gyrus/sulcus.

a [(PfSf + PfSc + PcSf + PcSc) − silence].

b [(PcSc + PcSf) − (PfSc + PfSf)].

c [(PcSc + PfSc) − (PcSf + PfSf)].

d [(PcSc − PcSf) − (PfSc − PfSf)].

## Appendix A Supplementary data

Audio 1

Audio 2

Audio 3

Audio 4
