# Supplementary material for: Functional neuroanatomy of spatial sound processing in Alzheimer's disease
Source: Neurobiol Aging. 2016 Mar;39:154–64. doi: 10.1016/j.neurobiolaging.2015.12.006 (PMC4782736; doi:10.1016/j.neurobiolaging.2015.12.006)
Supplement: Supplementary Material [file mmc5.docx]

# Supplementary material. Functional neuroanatomy of spatial sound processing in Alzheimer’s disease, by HL Golden et al

# Table S1. Summary of HRTF data in this study

| **HRTF I.D.** | **Gender** | **Height** (inches) | **No. this study** | |
| --- | --- | --- | --- | --- |
|  |  |  | **Healthy controls** | **AD** |
| SJX | F | 68.0" | 2 | 1 |
| SOU | F | 65.0" | 6 | 5 |
| SOS | M | 74.0" | 8 | 7 |
| SOW | M | 75.0" | 0 | 1 |

Background height and gender data (Wightman and Kistler, 1989) for individuals used to generate the generic head-related transfer functions (HRTFs) used in the current study; the number of participants for whom each HRTF was used is indicated [data from: Wightman FL, Kistler DJ. 1989. Headphone simulation of free-field listening. II: Psychophysical validation. J Acoust Soc Am. 85:868–878].

# Table S2. Regions of grey matter atrophy in the Alzheimer’s disease group

| **Region** | **Side** | **Cluster**  (voxels) | **Peak** (mm) | | | ***t*-value** |
| --- | --- | --- | --- | --- | --- | --- |
|  |  |  | **x** | **y** | **z** |  |
| Hippocampus/entorhinal cortex | R | 1198 | 35 | -12 | -39 | 5.91 |
| Hippocampus/amygdala | L | 602 | -29 | 2 | -26 | 5.82 |
| Inferior temporal gyrus | R | 568 | 47 | -4 | -38 | 5.73 |
|  | L | 158 | -50 | -28 | -18 | 5.08 |
| Inferior temporal sulcus/medial temporal gyrus | R | 118 | 65 | -33 | -15 | 4.50 |
| Inferior temporal gyrus | L | 515 | -60 | -55 | -11 | 5.01 |
|  | L | 115 | -48 | -6 | -38 | 4.17 |
| Lateral occipitoparietal cortex | L | 95 | -20 | -84 | 30 | 4.83 |
| Medial occipitoparietal cortex | L | 66 | -8 | -85 | 39 | 5.08 |
| Posterior cingulate cortex | R | 71 | 11 | -55 | 33 | 4.98 |
| Dorsolateral prefrontal cortex | R | 65 | 46 | 30 | 27 | 4.79 |
| Temporo-parietal junction | L | 56 | -45 | -54 | 25 | 4.01 |
| Middle temporal gyrus/superior temporal sulcus | R | 54 | 52 | -51 | 10 | 4.87 |

Regions of significant regional grey matter atrophy in the Alzheimer’s disease group compared with the healthy control group from the voxel-based morphometry analysis are presented (see also Figure 2). Associations shown are significant at threshold p < 0.001 uncorrected for multiple comparisons over the whole brain; all significant clusters > 50 voxels are shown and peak (local maxima) coordinates are in MNI space.

**Table S3. Additional whole-brain fMRI data for contrasts of interest in participant groups**

| **Group** | **Contrast** | **Region** | **Side** | **cluster**  (voxels) | **Peak** (mm) | | | **t-value** |
| --- | --- | --- | --- | --- | --- | --- | --- | --- |
|  |  |  |  |  | x | y | z |  |
| **HEALTHY CONTROLS** | Sound > silence^a^ | Inferior colliculus | R | 419 | 8 | -31 | -6 | 6.43 |
|  |  | Oribitofrontal cortex | L | 173 | -9 | 53 | -17 | 6.32 |
|  |  | Cerebellum | L | 86 | -24 | -64 | -53 | 5.99 |
|  |  | Cerebellum | R | 54 | 12 | -85 | -33 | 5.22 |
|  | Changing > fixed pitch^b^ | Premotor cortex | L | 62 | -17 | -7 | 61 | 7.35 |
|  |  | Mid STG/STS | L | 217 | -60 | -16 | 4 | 4.84 |
|  | Changing > fixed location^c^ | Inferior frontal gyrus | L | 166 | -60 | 9 | 27 | 7.12 |
|  |  | Superior parietal lobe | L | 226 | -45 | -22 | 60 | 6.84 |
|  |  | Premotor cortex | L | 69 | -53 | 3 | 42 | 6.63 |
|  |  | Premotor cortex | R | 59 | 52 | 8 | 46 | 6.16 |
|  |  | Inferior parietal lobe | L | 58 | -36 | -61 | 51 | 5.91 |
|  | Changing pitch vs. changing location^d^ | Posterior fusiform gyrus | R | 55 | 24 | -76 | -17 | 6.38 |
| **AD PATIENTS** | Sound > silence^a^ | Inferior colliculus | L | 567 | -11 | -33 | -9 | 8.92 |
|  |  | Angular gyrus | L | 51 | -57 | -27 | 34 | 6.82 |
|  |  | Cerebellum | R | 292 | 36 | -75 | -24 | 6.50 |
|  |  | Cerebellum | L | 124 | -24 | -60 | -18 | 5.97 |
|  | Changing > fixed pitch^b^ | Posterior STG | R | 59 | 66 | -25 | 7 | 4.56 |
| **AD > CONTROLS** | Sound > silence^a^ | Visual cortex | R | 108 | 5 | -90 | -3 | 4.56 |
|  |  | Premotor cortex | R | 75 | 39 | -16 | 36 | 4.01 |

The Table shows additional regional grey matter activations for contrasts of interest within each participant group and between groups, only revealed using a more lenient significance threshold p<0.001 uncorrected for multiple comparisons over the whole brain (compare Table 2 and Figure 3); clusters >50 voxels in size that comprised a unique locus are presented and peak (local maxima) coordinates are in MNI space. Contrasts were composed as coded by superscripts: **a,** [(PfSf + PfSc + PcSf + PcSc) – silence]; **b**, [(PcSc + PcSf) – (PfSc + PfSf)]; **c**, [(PcSc + PfSc) – (PcSf + PfSf)]; **d**, [(PcSc – PcSf) – (PfSc – PfSf)]. Conditions: PfSf = fixed pitch, fixed auditory spatial location; PcSf = changing pitch, fixed spatial location; PfSc = fixed pitch, changing spatial location; PcSc = changing pitch, changing spatial location. AD, Alzheimer’s disease; STG/STS, superior temporal gyrus/sulcus.

# Figure S1. Small volumes used for analysis of functional data


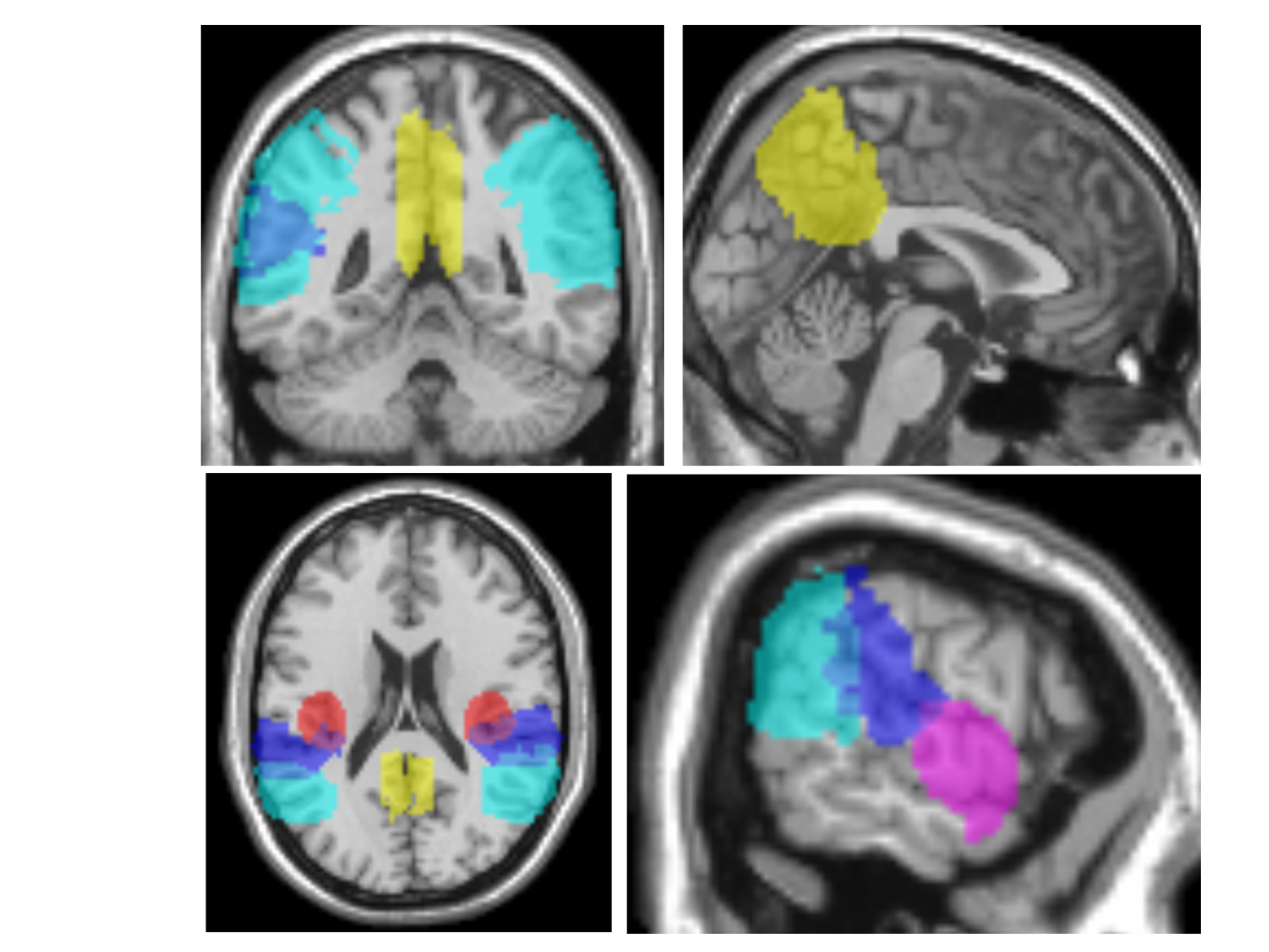


Representative slices illustrate the extent of the areas used to investigate voxel activity in small volumes. Yellow, PMC (edited Oxford-Harvard map); Red, insula (lg2 Jülich map); Cyan, angular gyrus (encompassing TPJ: Oxford-Harvard map); Blue, posterior STG/PT (edited Oxford-Harvard map); Magenta, anterior STG (Oxford-Harvard map).

# Figure S2. Functional neuroanatomy of auditory stimulation in the healthy control and Alzheimer’s disease groups


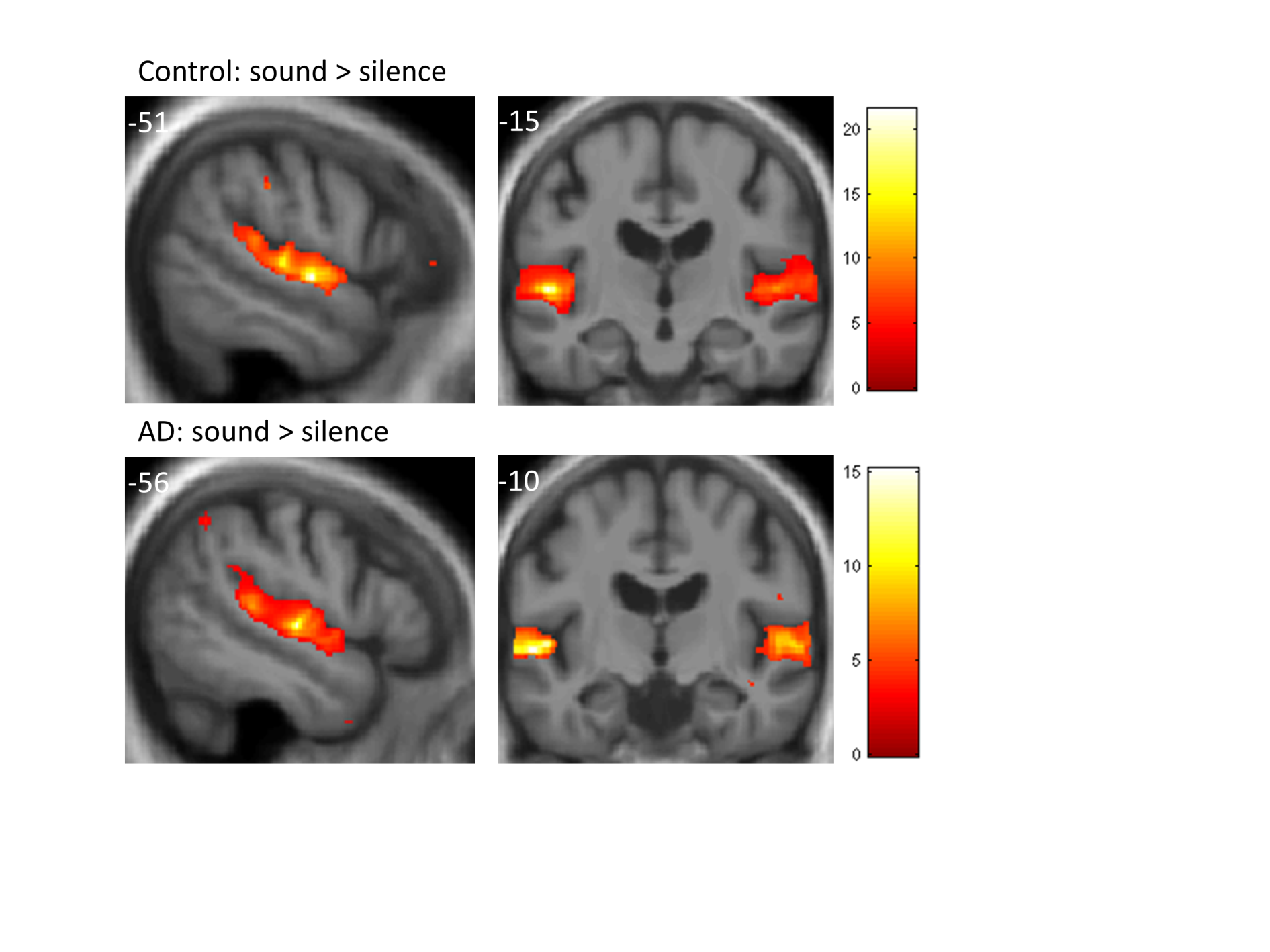


Statistical parametric maps show regions of greater activation for all sounds over silence [(PfSf + PfSc + PcSf + PcSc) – silence] for the healthy control (top panels) and Alzheimer’s disease (AD, bottom panels) groups. Clusters shown were significant at threshold p < 0.05 after correction for multiple comparisons within pre-specified anatomical regions of interest (see also Table I); however maps have been thresholded at p < 0.001 uncorrected over whole brain for display purposes. The colour side bars code voxel-wise t-values of grey matter activation. Planes of representative sections are indicated using the corresponding MNI coordinates (mm); the right hemisphere is shown on the right in the coronal sections.
